# The frequencies of naturally occurring protease inhibitor resistance mutations in HIV proviral sequences of drug naïve sex workers in Nairobi, Kenya and their correlation with host immune response driven positively selected mutations in HIV-1

**DOI:** 10.1186/1471-2334-14-S3-O2

**Published:** 2014-05-27

**Authors:** Raghavan Sampathkumar, Elnaz Shadabi, David La, John Ho, Binhua Liang, Joshua Kimani, Francis A Plummer, Ma Luo

**Affiliations:** 1 Department of Medical Microbiology, University of Manitoba, Winnipeg, Manitoba, Canada; 2 Department of Medical Microbiology, University of Nairobi, Nairobi, Kenya; 3 National Microbiology Laboratory, Public Health Agency of Canada, Winnipeg, Manitoba, Canada

## Background

Sub Saharan Africa accounts for 69% of the people living with HIV globally. Antiretroviral therapy (ART) has saved 9 million life years in Sub Saharan Africa. However, drug resistance mutations reduce the effectiveness of ART, and need to be monitored for effective ART. Naturally occurring primary antiretroviral drug resistance mutations have not been well analyzed in ART naïve HIV+ patients from Kenya.

## Methods

We examined protease inhibitor (PI) resistance mutations in ART naïve HIV-1 seropositive women from Pumwani sex worker cohort, established in Nairobi, Kenya, wherein HIV-1 infection is predominantly caused by subtypes A and D viruses. We have analyzed consensus sequences of HIV protease from 234 drug naïve patients, as a part of HIV-1 whole genome sequencing using 454 sequencing methodology.

## Results

Analysis using HIVdb program revealed a prevalence of 0.56% of PI resistance major mutations (1/178; D30N) and 19.1% PI resistance minor mutations (34/178) among the study subjects. D30N mutation, which occurred along with minor mutations G48K and G73S, is known to confer high level resistance to nelfinavir. Several minor mutations were found at five different drug resistance sites. Positive selection analysis and correlation with disease progression revealed L10I, a PI resistance minor mutation and a positively selected mutation driven by host immune response, to be detrimental to host.

## Conclusion

This study provides valuable data on primary drug resistance in Kenyan HIV-1 infected patients before ART became available as well as HLA mediated immune pressure over HIV-1 protease.

